# Therapeutic Gazette

**Published:** 1885-04

**Authors:** 


THE THERAPEUTIC GAZETTE.—This periodical is already so well established that we need not refer to its merits, and the February number well sustains its reputation. The articles "Therapeutic Evidence" and "Cocaine" will be found of special interest. Subscriptions at 1925 Chestnut street, Philadelphia; $2.00 per annum.

